# Pulmonary function test-related prognostic models in non-small cell lung cancer patients receiving neoadjuvant chemoimmunotherapy

**DOI:** 10.3389/fonc.2024.1411436

**Published:** 2024-06-25

**Authors:** Min Zhang, Liang Zhu, Sibei Liang, Zhirong Mao, Xiaolin Li, Lingge Yang, Yan Yang, Kai Wang, Pingli Wang, Weiyu Chen

**Affiliations:** ^1^ Department of Respiratory and Critical Care Medicine, The Second Affiliated Hospital of Zhejiang University School of Medicine, Hangzhou, China; ^2^ Department of Rheumatology, the Second Affiliated Hospital, Zhejiang University School of Medicine, Hangzhou, China; ^3^ Department of Respiratory and Critical Care Medicine, Center for Oncology Medicine, the Fourth Affiliated Hospital of School of Medicine, and International School of Medicine, International Institutes of Medicine, Zhejiang University, Yiwu, China; ^4^ Zhejiang Key Laboratory of Precision Diagnosis and Treatment for Lung Cancer, Yiwu, China; ^5^ Department of Nutrition, the Fourth Affiliated Hospital of School of Medicine, and International School of Medicine, International Institutes of Medicine, Zhejiang University, Yiwu, China

**Keywords:** non-small cell lung cancer, pulmonary function test, prognostic model, neoadjuvant therapy, chemoimmunotherapy

## Abstract

**Background:**

This study aimed to establish a comprehensive clinical prognostic risk model based on pulmonary function tests. This model was intended to guide the evaluation and predictive management of patients with resectable stage I-III non-small cell lung cancer (NSCLC) receiving neoadjuvant chemoimmunotherapy.

**Methods:**

Clinical pathological characteristics and prognostic survival data for 175 patients were collected. Univariate and multivariate Cox regression analyses, and least absolute shrinkage and selection operator (LASSO) regression analysis were employed to identify variables and construct corresponding models. These variables were integrated to develop a ridge regression model. The models’ discrimination and calibration were evaluated, and the optimal model was chosen following internal validation. Comparative analyses between the risk scores or groups of the optimal model and clinical factors were conducted to explore the potential clinical application value.

**Results:**

Univariate regression analysis identified smoking, complete pathologic response (CPR), and major pathologic response (MPR) as protective factors. Conversely, T staging, D-dimer/white blood cell ratio (DWBCR), D-dimer/fibrinogen ratio (DFR), and D-dimer/minute ventilation volume actual ratio (DMVAR) emerged as risk factors. Evaluation of the models confirmed their capability to accurately predict patient prognosis, exhibiting ideal discrimination and calibration, with the ridge regression model being optimal. Survival analysis demonstrated that the disease-free survival (DFS) in the high-risk group (HRG) was significantly shorter than in the low-risk group (LRG) (P=2.57×10^-13^). The time-dependent receiver operating characteristic (ROC) curve indicated that the area under the curve (AUC) values at 1 year, 2 years, and 3 years were 0.74, 0.81, and 0.79, respectively. Clinical correlation analysis revealed that men with lung squamous cell carcinoma or comorbid chronic obstructive pulmonary disease (COPD) were predominantly in the LRG, suggesting a better prognosis and potentially identifying a beneficiary population for this treatment combination.

**Conclusion:**

The prognostic model developed in this study effectively predicts the prognosis of patients with NSCLC receiving neoadjuvant chemoimmunotherapy. It offers valuable predictive insights for clinicians, aiding in developing treatment plans and monitoring disease progression.

## Introduction

Lung cancer, recognized globally as a primary malignant tumor, is treated with various mainstream methods, including chemotherapy, targeted therapy, immunotherapy, and surgery. These treatments can significantly improve patient prognosis when appropriately administered (1, 2). In particular, recent advancements in immunotherapy have significantly improved survival rates for lung cancer cases, especially for non-small cell lung cancer (NSCLC), which accounts for 80–85% of them (2). Clinical trials have demonstrated that immunotherapy significantly enhances the overall survival (OS) of patients with advanced NSCLC, with immunotherapy recipients experiencing up to five times longer survival than those receiving chemotherapy alone (3). For patients with resectable IB-IIIA NSCLC receiving neoadjuvant therapy, immunotherapy has shown superior efficacy over chemotherapy in achieving a major pathological response (MPR) (4).

Despite these advances, a persistent issue in clinical studies is the variability in individual responses to immunotherapy, posing a challenge in predicting patient outcomes and risks. Therefore, numerous studies have sought to identify reliable prognostic predictors and establish clinical risk models. Research employing tumor microenvironment, genomics–pathology correlation, and deep learning models has successfully predicted patient responses (5–7). However, the limited availability of these diagnostic tests in routine clinical settings hampers their widespread adoption.

In our research, patients with NSCLC who were receiving neoadjuvant chemoimmunotherapy were chosen as the research cohort, incorporating 68 clinical features and laboratory parameters, including pretreatment pulmonary function indicators and postoperative complete pathological response (CPR) and MPR rates, to identify seven prognostic markers. Comprehensive prognostic models were constructed utilizing these indicators to evaluate this patient group’s disease-free survival (DFS). This model aimed to identify patients at risk of shorter DFS, for whom additional adjuvant treatment options such as chemotherapy, radiotherapy, or targeted therapy may be beneficial in improving patient outcomes and prolonging survival. This research aimed to offer crucial insights for refining clinical treatment strategies and optimizing patient care.

## Patients and methods

### Data collection and processing

This study meticulously gathered data from 175 patients with stage I-III NSCLC who underwent preoperative neoadjuvant chemoimmunotherapy at the Second Affiliated Hospital of Zhejiang University School of Medicine from December 1, 2018, to July 30, 2022. The immunotherapy regimen included programmed cell death protein 1/programmed death-ligand 1 antibodies (anti-PD1/PD-L1) such as pembrolizumab, nivolumab, durvalumab, tislelizumab, camrelizumab, sintilimab, and toripalimab. Chemotherapy was personalized by clinicians according to the pathology, involving the use of pemetrexed, paclitaxel, or protein-bound paclitaxel in combination with a platinum-based drug (carboplatin, cisplatin, nedaplatin) over 2–4 cycles of neoadjuvant therapy. A multi-disciplinary team consulted for each patient to evaluate suitability for operation. Patient conditions were closely monitored postoperatively for their status. Adjuvant treatment was tailored to individual patient conditions.

The principal inclusion criteria included: A) A preoperative histopathological diagnosis of primary NSCLC. B) Classification as surgically resectable stages I-III according to the 8th edition of the American Joint Committee on Cancer (AJCC) lung cancer staging criteria. C) Age >18 years. D) Initiation of at least one cycle of combined immunotherapy and chemotherapy post-diagnosis in our hospital. E) Eligibility for operation after neoadjuvant treatment and completion of the surgical procedure in our hospital with a minimum of 2 months follow-up. F) Availability of comprehensive baseline clinical data and test results. G) A performance status (PS) score of 0 or 1.

The exclusion criteria were: A) A history of severe immune deficiency, including positive human immunodeficiency virus (HIV) tests or organ transplantation. B) Diagnosis of other cancers within the past 5 years. C) Incomplete postoperative imaging data for evaluation. D) Death due to surgical complications.

Clinical pathological characteristics and prognostic survival data were retrieved from the electronic medical records. After treatment, MPR was characterized by ≤10% viable tumor cells in the surgical resection specimen. CPR was identified by the complete absence of viable tumor cells in the resected tissue upon pathological examination post-treatment. DFS was measured from the surgical date to the disease progression or the last follow-up, concluding on September 30, 2022, with outpatient and telephone follow-ups employed. This study received approval from the Institutional Review Board of the Second Affiliated Hospital of Zhejiang University School of Medicine (Approval No.: IR2022396).

### Univariate and multivariate Cox regression analysis

Survival analyses were conducted using the “survival” package (version 3.3–1) (8) in R 4.2.2 to ascertain the variables impacting patients’ OS. Variables with P< 0.1 were selected for a multivariate Cox regression analysis. Survival curves were generated using the “ggsurvplot()” function of the “survminer” package (version 0.4.9) (9). The optimal cutoff values for continuous variables were determined using the “surv_cutpoint()” function, categorizing them into high-risk (HRG) and low-risk groups (LRG) accordingly.

Subsequently, multivariable Cox regression analysis was performed using the “coxph()” and “step()” functions with a “backward” direction. Variables with P< 0.05 were ultimately chosen for the Cox regression model construction. The selected variables, along with their 95% confidence intervals (CIs), hazard ratios (HRs), and P-values, were plotted using the “forestplot” package (version 1.0.0) (10).

### Least absolute shrinkage and selection operator regression analysis

The “glmnet” package (11, 12) (version number: 4.1–6) was utilized to construct the LASSO regression model, incorporating variable data and patients’ survival information. Settings were adjusted as follows: the family parameter was set to “cox,” “alpha” to 1, and “nfolds” to 10. The optimal value of λ, minimizing the partial likelihood deviation, was selected. This optimal λ value was substituted in the “coef()” function to obtain the regression coefficients for each variable, facilitating the construction of the LASSO regression model.

### Ridge regression analysis

Variables identified as significant from the univariate Cox regression analysis were integrated with those used in the LASSO regression model. The results were plotted using the “Vennerable” package (version 3.0) (13) through a Venn diagram. Subsequently, these variables, along with patient survival data, were analyzed using the “glmnet” function with family set to “cox,” “alpha” to 0, and “nfolds” to 10. The “cv. glmnet()” function helped observe changes in partial likelihood deviation across different λ values. The identified optimal λ was used in the “coef()” function to calculate each variable’s regression coefficients for constructing the ridge regression model. Moreover, a Sankey diagram was created using the online platform SangerBox (http://vip.sangerbox.com/), illustrating variables and their classifications (risk or protective factors) from the three mentioned models (14).

### Risk score calculation and model evaluation

Risk scores for the three models were calculated using the “predict()” function of the “survival” package (8), with cutoff values determined by the “surv_cutpoint()” function from the “survminer” package (9). Patients were subsequently classified into HRG and LRG based on these scores, integrating this information into their profiles for subsequent analysis. The formulas for calculating risk scores were as follows:


$$\begin{array}{l} Cox-associated\;risk\;score\;(CARS)\\ \;\;=h_{0}(t)\times exp(\beta_{1}x_{1}+\beta_{2}x_{2}+\cdots +\beta_{n}x_{n}) \end{array}$$

$$LASSO-associated\;risk\;score\;\left(LARS\right)=\beta_{1}x_{1}+\beta_{2}x_{2}+\cdots +\beta_{n}x_{n}$$

$$Ridge-associated\;risk\;score\;\left(RARS\right)=\beta_{1}x_{1}+\beta_{2}x_{2}+\cdots +\beta_{n}x_{n}$$

In the above formula, x is the variable value, β is the regression coefficient, and n is the number of variables.

Risk factor correlation diagrams were drawn based on these scores, patient survival status, survival time, and variable values. Model calibration was assessed through calibration curves plotted using the “calibrate()” function of the “rms” package (version 6.5.0) (15). Nomograms for risk scores were also plotted to check for linear fitting.

The prognostic capability of the models was evaluated by plotting survival curves for the different risk groups and survival periods, calculating the area under the curve (AUC) values at three DFS time points (1 year, 2 years, and 3 years) using the “timeROC” package (version 0.4) (16), and drawing receiver operating characteristic (ROC) curves. Precision recall (PR) capabilities for predicting clinical outcomes were assessed using the “modEvA” package (version 3.9.3) (17) to plot PR curves (PRC) and calculate AUC values.

The net reclassification improvement (NRI) and integrated discrimination improvement (IDI) between the Cox, LASSO, and ridge regression models were calculated using the “survIDINRI” package (version 1.1–2) (18). Lastly, the clinical net benefit of the three models at the three DFS time points was evaluated using the “ggDCA” package (version 1.2).

### Internal verification and model comparison

Without external validation cohorts, internal validation was conducted on these models. Initially, the cohort was randomly divided into training and validation sets at a ratio of 1:1. Subsequently, the “cph()” function from the “rms” package (15) was employed to fit both sets based on the risk scores of the three models. The models’ discrimination and goodness of fit were assessed using the “Cindex()” and “calPlot()” functions from the “pec” package (version 2022.05.04) (19), respectively.

### Clinical relevance analysis of the optimal model

The clinical baseline data were integrated with the risk scores and risk group categorizations derived from the optimal model. Factors not incorporated in the model underwent correlation analysis to explore their potential clinical relevance. For categorical variables, such as gender and pathological type, analyses were performed to identify significant differences in risk scores across different baseline groupings. Similarly, the analyses determined differences between the HRG and LRG for continuous variables, such as age and height. The correlation between continuous variables and risk scores was analyzed to evaluate the potential clinical applicability of the optimal model.

### Statistical analysis

The study’s data was processed using R 4.2.2 and GraphPad Prism (version 9.0.0, San Diego, California, USA) software. The “ggplot2” package (version 3.3.5) (20) was employed for data visualization, adhering to default parameters for unspecified methods. Continuous variables were presented as mean ± standard deviation (SD). The Mann–Whitney U test was applied to non-normally distributed data, while the Student’s t-test was utilized to compare two groups with normally distributed and equal variance data. For data with normal distribution but unequal variances, Welch’s t-test was used. Categorical variables were described using frequencies and percentages, with the chi-square test used for group comparisons. Spearman’s correlation test was conducted for correlation analyses, and the “ggpubr” package (version 0.6.0) (21)was used for the statistical analysis of scatter plots. Additional histograms were created with “ggExtra” (version 0.10.0) (22). Statistical significance was set at P < 0.05, with * indicating P < 0.05, ** P < 0.01, *** P < 0.001, and **** P < 0.0001.

## Results

### Baseline characteristics of patients

The study included 175 patients, comprising 20 (11.4%) women and 155 (88.6%) men, aged 40–81 years, with a median age of 65 years. A total of 61 (34.9%) patients had no history of smoking, while 114 (65.1%) were smokers. Lung squamous cell carcinoma (LUSC) was diagnosed in 123 (70.3%) patients, with the remaining 52 (29.7%) presenting other pathologies. Staging, according to the eighth edition of the AJCC, included six (3.4%) patients in stage I, 53 (30.3%) in stage II, and 116 (66.3%) in stage III. Tumor stages were T1, T2, T3, and T4 in 20 (11.4%), 77 (44.0%), 53 (30.3%), and 25 (14.3%) patients, respectively, with nodal stages N0, N1, N2, and N3 in 32 (18.3%), 51 (29.1%), 91 (52.0%), and one (0.6%) patient, respectively.

All participants underwent preoperative neoadjuvant chemoimmunotherapy, with two (1.1%) receiving one course, 130 (74.3%) receiving two, 36 (20.6%) receiving three, and seven (4.0%) receiving four. MPR was achieved by 95 (54.3%) patients and CPR by 67 (38.3%). A total of 158 patients (90.3%) received postoperative adjuvant therapy, and 17 patients (9.7%) did not undergo further treatment. The baseline data and the results of the univariate Cox regression analysis are shown in **Supplementary Table S1**.

**Figure 1:**
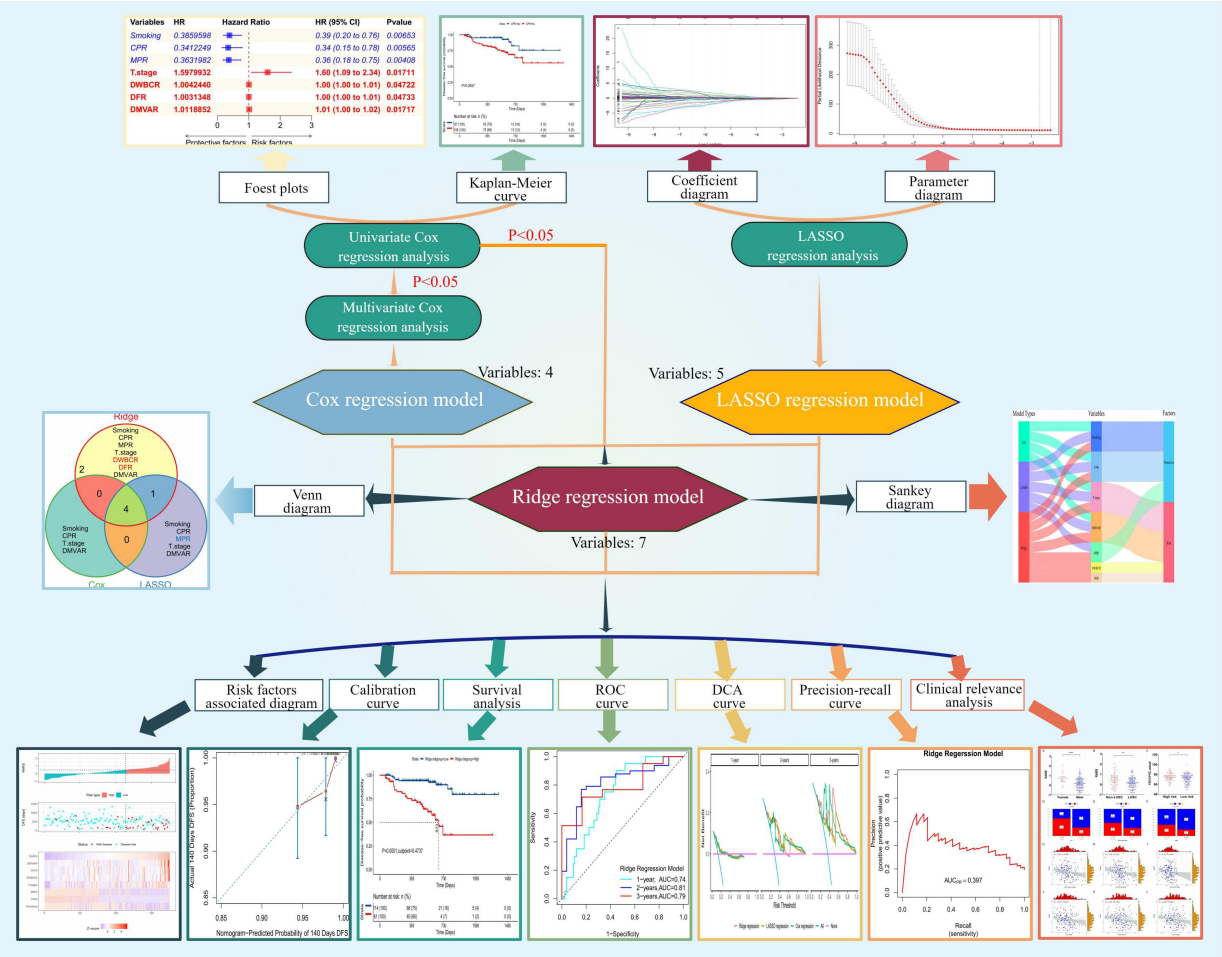
Flowchart of the study.

The flowchart of this study is illustrated in **Figure 1**, created using Figdraw (https://www.figdraw.com/).

### Cox regression model constructed with four variables

**Figure 2:**
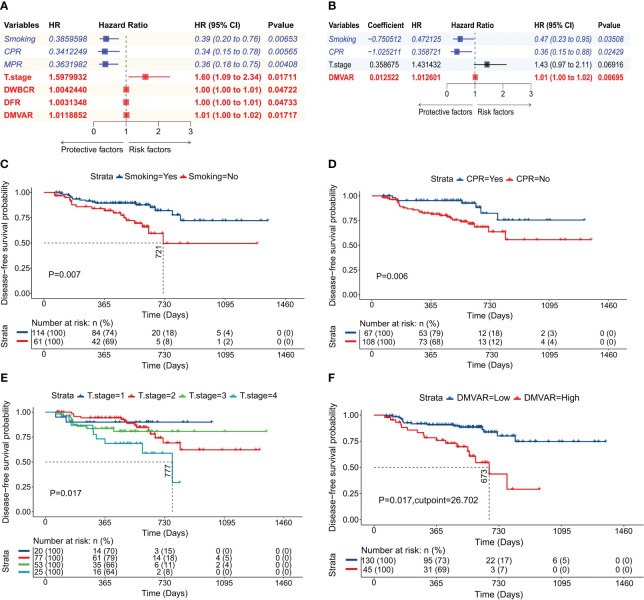
Construction of the Cox regression model. **(A)** Forest plot of univariate Cox regression analysis detailing variables along with their HR, 95% CI, and P values; **(B)** Forest plot of multivariate Cox regression analysis showing variables, regression coefficients, HR, 95% CI, and P values; **(C)** Survival curve based on smoking status; **(D)** Survival curve based on CPR achievement; **(E)** Survival curve according to T stage; **(F)** Survival curve derived from the optimal cutoff value of DMVAR (DMVAR=26.702).

Univariate Cox regression analysis identified seven variables impacting DFS among 68 studied variables. The hazard ratios (HR), 95% confidence intervals (CI), and P values for these variables are shown in **Figure 2A**. Subsequently, multivariate Cox regression analysis refined these to four significant variables, as illustrated in **Figure 2B**. Smoking (P=0.007, **Figure 2C**) and CPR (P=0.006, **Figure 2D**) were identified as protective factors. In contrast, the T stage (P=0.017, **Figure 2E**) and the D-dimer/minute ventilation volume actual ratio (DMVAR) (P=0.017, **Figure 2F**) were recognized as risk factors.

A regression model was developed based on regression coefficients. The formula for calculating the risk score in this model was:


$$\begin{array}{l} CARS=-1.197433\times exp(-0.750512\times Smoking\;value\\ \;\;\;-1.025211\times CPR\;value+0.358675\times T\;stage\;value\\ \;\;\;+0.012522\times DMVAR\;value) \end{array}$$

In the above formula, smoking history was indicated by 1 (presence) or 0 (absence); the attainment of CPR was characterized by 1 (achieved) or 0 (not achieved), and the T stage was represented by values 1, 2, 3, or 4 corresponding to T1, T2, T3, or T4 stages, respectively.

### Construction of the LASSO regression model using five variables

**Figure 3:**
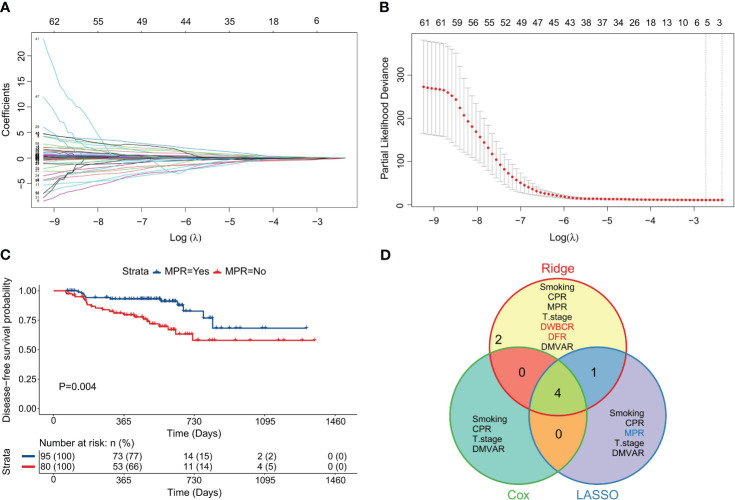
Construction of the LASSO regression model. **(A)** Diagram illustrating the distribution of LASSO regression coefficients, showcasing the variation in regression coefficients (ordinate) against log(λ) (abscissa) under 10-fold cross-validation. **(B)** Graph depicting LASSO regression parameters, highlighting the trend of partial likelihood deviation (ordinate) with log(λ) (abscissa) under 10-fold cross-validation; vertical dashed lines represent the optimal λ value using the minimum criterion and one standard error from the minimum; **(C)** Survival curve based on MPR achievement; **(D)** Venn diagram illustrating the variables included across the three models.


LASSO regression was applied to all 68 variables, employing the minimized λ and a 10-fold cross-validation method to identify five variables with non-zero regression coefficients (**Figure 3A**). As illustrated in **Figure 3B**, the model had the lowest partial likelihood deviation when λ was at its minimum, leading to the construction of a LASSO regression model based on the regression coefficients of five variables, with the risk score formula being:

$$\begin{array}{c} LARS=-0.202190\times Smoking\;value-0.057328\times CPR\;value\\ \;\;\;-0.225867\times MPR\;value+0.046980\times T\;stage\;value\\ \;\;\;+0.004285\times DMVAR\;value \end{array}$$

The classification for variables remained consistent with that in CARS. MPR, a protective factor, was assigned a value of 1 (achieved) or 0 (not achieved) (P=0.004, **Figure 3C**).

### Construction of the ridge regression model using seven variables

**Figure 4:**
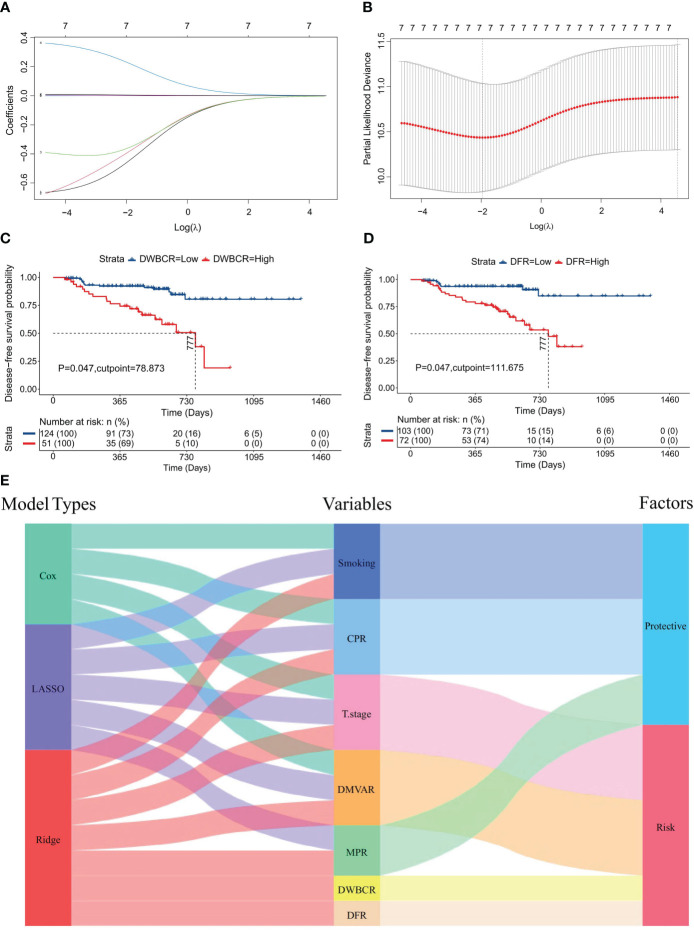
Construction of the ridge regression model. **(A)** Distribution diagram of ridge regression coefficients, illustrating changes in coefficients (ordinate) against log(λ) (abscissa) within 10-fold cross-validation; **(B)** Ridge regression parameter graph, showing the likelihood deviation trends (ordinate) with log(λ) (abscissa) under cross-validation; **(C)** Survival curve derived from the optimal DWBCR cutoff value (DWBCR=78.873). **(D)** Survival curve from the optimal DFR cutoff value (DMVAR=111.675). **(E)** Sankey diagram reflecting the integration of variables and their roles across the three regression models.


A ridge regression model was developed by using seven variables identified from univariate analysis. The overlap of variables across the three models is visualized in **Figure 3D**. The minimum λ value was determined by employing the minimization of λ through 10-fold cross-validation, as shown in **Figure 4A**. **Figure 4B** demonstrates the model’s lowest partial likelihood deviation at this λ value. Regression coefficients for each variable were calculated at this λ, leading to the following risk formula:

$$\begin{array}{l} CARS=-0.454011\times Smoking\;value-0.389617\times CPR\;value\\ \;\;\;-0.360084\times MPR\;value+0.227656\times T\;stage\;value\\ \;\;\;+0.000871\times DWBCR\;value+0.001024\times DFR\;value\\ \;\;\;+0.005581\times DMVAR\;value \end{array}$$

In the above formula, the assignment of variables mirrors that in CARS or LARS. Variables with positive coefficients in the formula were suggested as risk factors, whereas those with negative coefficients were deemed protective. Among them, the D-dimer/white blood cell ratio (DWBCR) (P=0.047, **Figure 4C**) and the D-dimer/fibrinogen ratio (DFR) (P=0.047, **Figure 4D**) were identified as risk factors. **Figure 4E** displays the variables and their classifications as factors in the three models.

### All three models could effectively predict patient outcomes with optimal discrimination and calibration

**Figure 5:**
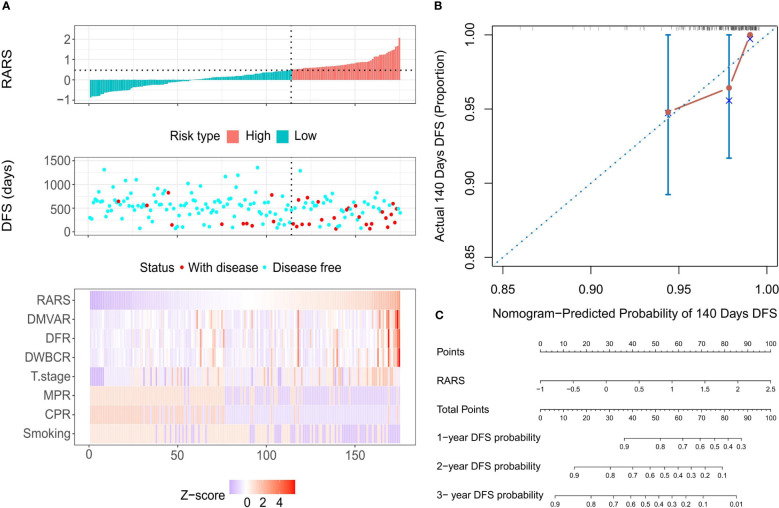
Calibration evaluation of the Ridge regression model. **(A)** Risk factor correlation diagram featuring histograms of the distribution of patients’ risk scores, scatter plots of patient survival distributions, and heatmaps showing changes in variables with risk scores. The horizontal axis represents patient numbers, ranked from lowest to highest risk score; the vertical axis shows patients’ risk scores, DFS, and variables included in the model. **(B)** Calibration curve at the optimal calibration time point of 140 days, displaying predicted vs. actual survival status. Every 50 patients were grouped and resampled 1000 times. **(C)** Nomogram based on patients’ risk scores, showing total scores and the probability of 1-year, 2-year, and 3-year DFS.

By calculating the CARS, LARS, and RARS for each patient and correlating these with survival status and variable values, the prognostic prediction for the 175 patients was made using risk factor correlation diagrams (**Supplementary Figure S1A**). Subsequently, patients were organized by ascending CARS scores, with the optimal cutoff value of 0.334, segregating patients into HRG and LRG. The HRG showed a significantly higher mortality rate than the LRG, with high-risk factor values predominating in the HRG and protective factors in the LRG. Similar patterns were observed when patients were divided using the optimal cutoff values LARS=-0.062 (**Supplementary Figure S1B**) and RARS=0.4737 (**Figure 5A**), leading to comparable conclusions. Moreover, all three models exhibited precise fitting accuracy. Model calibration diagrams at the 140-day mark revealed optimal fitting; however, the goodness of fit (GOF) of the Cox regression model (**Supplementary Figure S1C**) was slightly inferior to that of the LASSO (**Supplementary Figure S1D**) and ridge regression models (**Figure 5B**). The nomograms show that the Cox (**Supplementary Figure S1E**), LASSO (**Supplementary Figure S1F**), and ridge regression models (**Figure 5C**) all had an optimal linear fitting, with risk scores linearly correlating with the nomogram’s total score, indicating prognostic prediction value.

**Figure 6:**
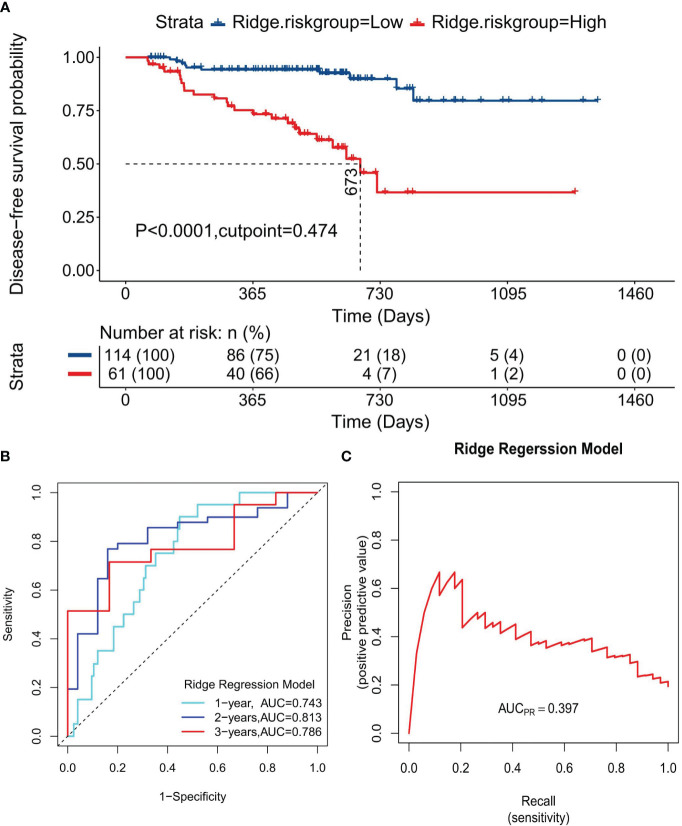
Discrimination evaluation of the Ridge regression model. **(A)** The survival curve plotted based on the optimal cutoff values of 0.042. **(B)** The ROC curve at 1-, 2-, 3-year disease-free survival time points, respectively. **(C)** PRC curve for the normalized risk scores of the ridge regression model.

Survival curves were plotted for HRG and LRG based on the risk stratification to evaluate the models’ prognostic prediction and discrimination capabilities. The median DFS for HRG in the Cox regression model was 777 days, significantly shorter than that of LRG, where the median DFS was not reached, indicating a significant difference between the two groups (P =6.32×10^-6^, **Supplementary Figure S2A**). Similarly, the median DFS for HRG in the LASSO regression model was 721 days, significantly shorter than that of the LRG, indicating a significant difference between the two groups (P=1.77×10^-6^, **Supplementary Figure S2B**). The ridge regression model followed this pattern, with HRG’s median DFS at 673 days, significantly shorter than that of the LRG, indicating a significant difference (P=2.57×10^-13^, **Figure 6A**). Time-dependent ROC curves revealed that the AUC values for the Cox regression model at 1-, 2-, and 3-year time points were 0.756, 0.807, and 0.825, respectively (**Supplementary Figure S2C**); 0.725, 0.811, and 0.723, for the LASSO regression model, respectively (**Supplementary Figure S2D**), and 0.743, 0.813, and 0.786, for the ridge regression model, respectively (**Figure 6B**). Moreover, PRCs were plotted to analyze the models’ accuracy in identifying patients with the disease, showing an increase in disease identification probability from 19.4% (34/175) to 38.6% (**Supplementary Figure S2E**), 37.9% (**Supplementary Figure S2F**), and 39.7% (**Figure 6C**) for the Cox, LASSO, and ridge regression models, respectively. These results suggest that all three models offer excellent discrimination and can predict patient prognosis and survival reliably.

### The ridge regression model emerged as the optimal model for this study

**Figure 7:**
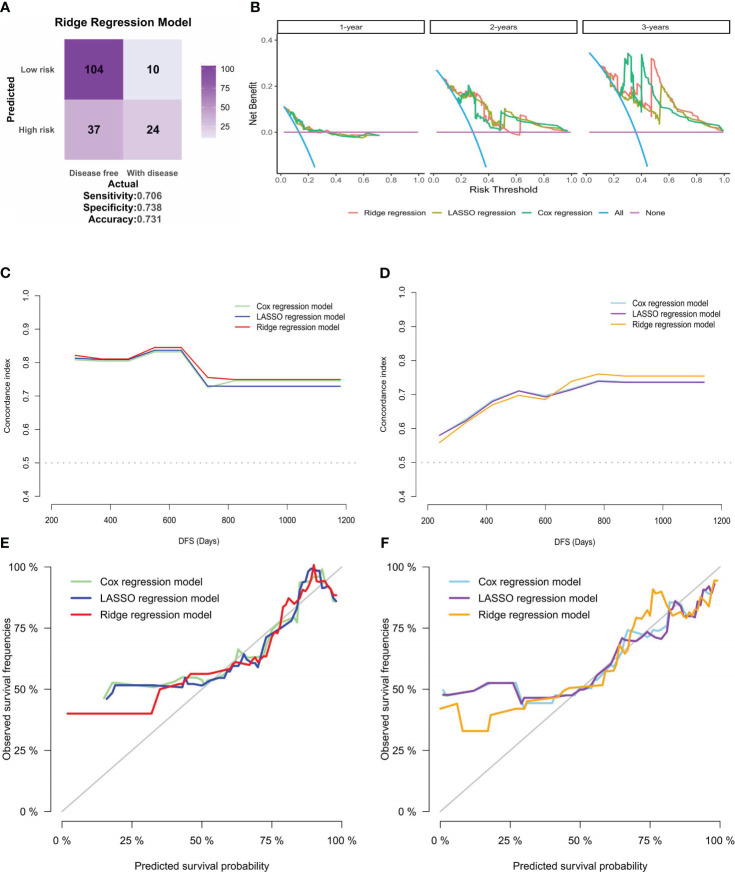
Clinical applicability and internal validation of models. **(A)** Confusion matrix for the ridge regression model, created based on the patients’ risk groups and their actual recurrence or death outcomes. **(B)** DCA for the three models at 1-year, 2-year, and 3-year intervals, with the risk threshold on the horizontal axis and net benefit on the vertical axis. **(C, D)**. Plots of C-statistics (vertical axis) versus patients’ DFS (horizontal axis) for the three models in the **(C)** training and **(D)** validation sets of internal validation; **(E, F)**. Calibration curves for the three models in the **(E)** training and **(F)** validation sets, displaying predicted versus actual survival probabilities.

To further establish the superiority of the ridge regression model over the other two models, the accuracy of the three models was evaluated using classifiers. The accuracies for the Cox and the LASSO regression models were 57.1% (**Supplementary Figure S2G**) and 63.4% (**Supplementary Figure S2H**), respectively. These values were surpassed by those of the ridge regression model, which achieved an accuracy of 73.1% (**Figure 7A**). Moreover, decision curve analysis (DCA) (**Figure 7B**) results indicated that all three models could enhance net benefits at the 1-, 2- and 3-year time points, with the ridge regression model showing a slight increase in net benefit compared to the other models. A thorough comparison across the models revealed that the ridge regression model consistently outperformed the others, at the 1-year, 2-year, and 3-year benchmarks, regardless of IDI, continuous NRI, or median improvement. This finding suggests that the ridge regression model had a superior predictive capability for outcome events in our study, as shown in **Supplementary Table S2**.

Due to the absence of external validation cohorts, internal validation was performed for the three models. The dataset was randomly partitioned into training and validation sets at a 1:1 ratio. Subsequently, the variation in C-statistics over survival time was charted. While the C-statistics for all three models displayed similarity, the ridge regression model exhibited superior discrimination, which was evident in training (**Figure 7C**) and validation sets (**Figure 7D**). Subsequently, the calibration curves for the three models were plotted for the training (**Figure 7E**) and validation sets (**Figure 7F**). The outcomes indicated that the ridge regression model exhibited superior GOF. Consequently, the ridge regression model emerged as the optimal model in this study based on the comprehensive evaluation and analysis of the models. It demonstrated the best discrimination and GOF, offering a more precise prediction of patients’ DFS in our cohort.

### Correlation of ridge regression model with multiple clinical prognostic factors

**Figure 8:**
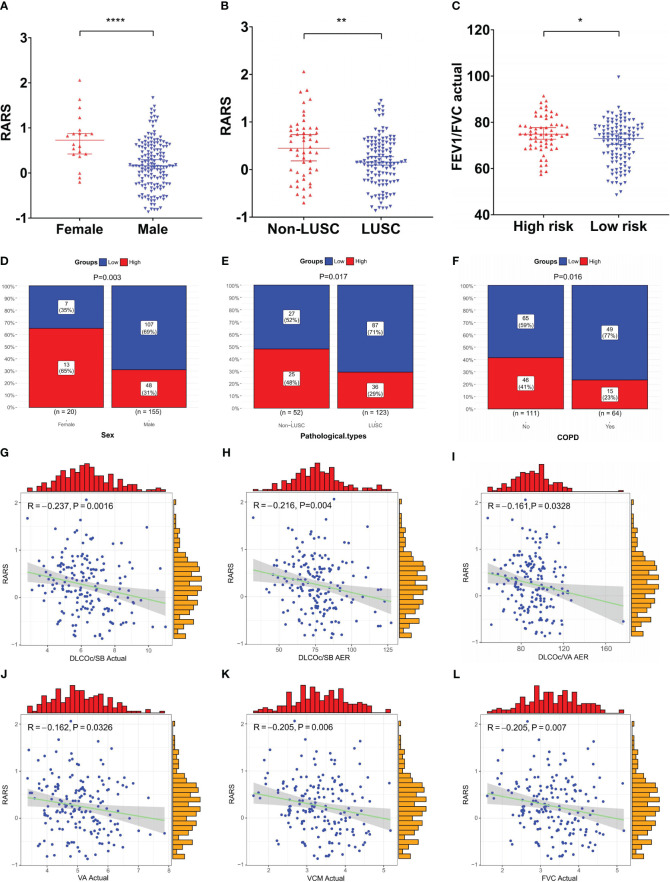
Clinical relevance analysis of the ridge regression model. **(A–C)** Significant differences in RARS were observed across **(A)** gender and **(B)** pathological types; **(C)** Significant differences in actual FEV1/FVC were observed between risk groups; **(D–F)** Significant disparities in risk groups were noted based on **(D)** gender, **(E)** pathological type, and **(F)** COPD status; **(G–L)** Significant negative correlations existed between RARS and pulmonary function indicators, **(G)** actual DLCOc/SB, **(H)** DLCOc/SB AER, **(I)** DLCOc/VA AER, **(J)** actual VA, **(K)** actual VCM, and **(L)** actual FVC. *P< 0.05, **P< 0.01, and ****P< 0.0001.

To further explore the clinical utility of the ridge regression model, this study investigated its correlation with various clinical factors not initially included in the model. The analysis focused on significant differences in RARS across genders, stages, and other clinical factors. Significant variations in RARS were observed across genders (P<0.0001, **Figure 8A**) and different pathological types (P=0.004, **Figure 8B**). Significant differences in the actual forced expiratory volume in one second/forced vital capacity (FEV1/FVC) (P=0.028, **Figure 8C**) were found between different risk groups. Similarly, a considerably higher incidence was noted among male patients in LRG (P=0.003, **Figure 8D**), those with LUSC (P=0.017, **Figure 8E**), and individuals with comorbid chronic obstructive pulmonary disease (COPD) (P=0.016, **Figure 8F**), suggesting these groups are more likely to exhibit a favorable prognosis.

Subsequently, the correlation between continuous variables, such as age and pulmonary function indicators, and RARS, was compared and analyzed. The findings revealed significant negative correlations between RARS and six pulmonary function indicators: actual diffusing capacity of the lung for carbon monoxide corrected for hemoglobin concentration per single breath (DLCOc/SB) actual (R = -0.237, P = 0.0016, **Figure 8G**), actual/estimate ratio (AER) of DLCOc/SB (R = -0.216, P = 0.004, **Figure 8H**), AER of DLCOc/alveolar volume (VA) (R = -0.161, P = 0.0328, **Figure 8I**), actual VA (R = -0.162, P = 0.0326, **Figure 8J**), actual vital capacity max (VCM) (R = -0.205, P = 0.0064, **Figure 8K**), and actual FVC (R = 0.205, P = 0.0066, **Figure 8L**). These results further underscore the prognostic predictive value of the model.

## Discussion

Patients with early-stage resectable NSCLC can benefit significantly from surgical treatment. However, certain patients with NSCLC may require neoadjuvant therapy to qualify for surgery (23). Recent advancements in immunotherapy have demonstrated that combining it with neoadjuvant therapy offers more benefits than purely neoadjuvant chemotherapy. Rosner et al. (24) discovered that achieving a postoperative MPR postoperatively can result in a 5-year DFS rate of up to 89%. MPR is associated with extended event-free survival (EFS) and OS (25, 26). However, relying on the postoperative pathological response rate as a single indicator has limitations. Clinical practice has observed that even among patients with identical clinical stages and similar treatment protocols, responses to treatment, postoperative efficacy, and prognostic outcomes vary. This variability could stem from the combined effects of individual factors, tumor heterogeneity, the immune microenvironment, PD-L1 expression, and tumor mutation burden (TMB) (27). Therefore, effective prediction models must be constructed to navigate these complexities.

Most existing studies on the prognosis of neoadjuvant therapy rely on bioinformatics analysis. For instance, Pang et al. (28) constructed a predictive model by analyzing immune-related differentially expressed genes. Ouyang et al. (29) constructed a risk prognostic model for LUAD by analyzing hypoxia-, immunity-, and epithelial-to-mesenchymal transition-related genes. However, these indicators are frequently challenging to measure in routine clinical settings. In contrast, pulmonary function tests provide critical insights into lung ventilation and diffusion functions, and are relatively easy to obtain. However, the application of pulmonary function tests in predicting the outcomes of NSCLC immunotherapy remains underexplored. This study pioneers prognostic models based on pulmonary function tests performed before the treatment, incorporating 68 clinical variables potentially influencing patient prognosis and survival. With DFS as the primary endpoint, predictive models were developed, encompassing a broad spectrum of factors, including individual drug treatments, surgical interventions, chronic conditions, and markers of coagulation and inflammation. After a thorough evaluation, seven indicators were identified as significant for the prognosis of patients with stage I-III NSCLC undergoing neoadjuvant chemoimmunotherapy. The constructed models demonstrated commendable discrimination, GOF, and accuracy, suggesting substantial clinical applicability.

T staging emerged as a crucial risk assessment indicator within our predictive framework. Universally acknowledged as a critical metric for evaluating the primary tumor’s size and extent of invasion, T staging holds paramount importance, especially for surgical candidates (7, 30). The application of T staging to predict postoperative survival in patients with early NSCLC is well-recognized. Wang et al. (7) demonstrated that patients with stage I NSCLC tumors measuring<20 mm in diameter exhibit improved postoperative prognoses. Sayan et al. (31) identified that a tumor size >21.5 mm significantly predicts occult lymph node metastasis in patients with stage IA NSCLC. Similar to neoadjuvant therapy, neoadjuvant immunotherapy aims to shrink the primary tumor and enable downstaging, thereby facilitating or simplifying surgery (32). Despite the variety of clinical prognostic prediction models for neoadjuvant therapy, T staging is commonly included and recognized as a critical predictor of patient outcomes (33, 34), aligning with the findings of our model.

Smoking stands as a primary independent risk factor for various types of cancer, accounting for up to 20% of all cancer cases in the United States, and is regarded as the most significant risk factor for cancer (35). Contrary to this general risk association, our research identified a history of smoking as a protective and prognostic factor in neoadjuvant chemoimmunotherapy, a conclusion supported by the findings of a meta-analysis (36). Furthermore, Li et al. (37) observed that among patients with LUAD and positive PD-L1 expression, those with over a year of smoking history showed an 85.2% survival rate post-immunotherapy, compared to 56.1% for former smokers and 42.6% for individuals with no smoking history. Another study highlighted that in the absence of TMB, smoking intensity could serve as a clinical predictor for immunotherapy effectiveness (38). Wang et al. (39) reported that tobacco smoke triggers PD-L1 expression in lung epithelial cells via the aryl hydrocarbon receptor (AhR), facilitating immune evasion and tumorigenesis. Experiments with mouse models have confirmed that AhR inhibitors significantly inhibit tumor growth and synergize with PD-L1 antibodies. To the best of our knowledge, smoking is a known risk factor for chronic COPD and lung cancer. Our study’s clinical correlation analysis indicated that patients with COPD, akin to those with a history of smoking, may experience longer DFS. This finding contradicts our previous understanding of lung cancer chemotherapy, where COPD was generally seen as a factor that significantly worsens the prognosis of patients receiving chemotherapy (40). In this context, Mark et al. (41) suggested that COPD subtypes might influence immune characteristics, with a significant increase in CD4+ cell proportion and Th1 polarization in COPD potentially underlying the enhanced response to immune therapy. Our analysis of pretreatment pulmonary function test data revealed that patients in the LRG exhibited lower FEV1/FVC ratios, correlating with a more favorable prognosis. Therefore, a history of smoking, FEV1/FVC ratios, and COPD presence were closely aligned with our study findings, suggesting that patients with a history of smoking history might fare better with neoadjuvant immunotherapy combined with chemotherapy, particularly those in the LRG who tend to have lower FEV1/FVC values and were more likely to have COPD comorbidities.

Our research found chemoimmunotherapy to be more effective in male patients or those with LUSC, which emerged as significant prognostic indicators. Previous research has highlighted the influence of gender on the efficacy of immunotherapy across various cancer types, with male patients frequently deriving more benefits (42). Pinto et al. (43) observed that while overall mortality risk for NSCLC decreased in women, men experienced a 24% reduction in risk with PD-L1 inhibitor therapy, unlike women, who did not show significant improvement. This disparity might stem from the complexity of immune expression in women, who typically exhibit more robust innate and adaptive immune responses (44). In our model’s clinical baseline data, LUSC was predominantly found in men (77.4%), whereas a higher proportion of women had other pathological types (85.0%); this difference was significant (P<0.0001). The variation in pathological types affects the prognosis of neoadjuvant therapy for NSCLC, with Faruki et al. (45) revealing significant differences in the molecular expression subtypes between LUSC and LUAD regarding the immune host response, potentially explaining the varied reactions to immunotherapy. Gao et al. (46) observed a significant difference in the MPR rates between LUSC and LUAD during immunotherapy with sintilimab (48.4% vs 0%). Moreover, a multicenter study on neoadjuvant chemoimmunotherapy revealed that patients with LUSC achieved an MPR of 80%, significantly higher than the 53% observed in those with LUAD (47). However, LUAD has a higher likelihood of epidermal growth factor receptor (EGFR) mutations, especially among the Asian population, where the incidence is approximately 50% (48), necessitating alternative treatment strategies. Therefore, the differential response to immunotherapy in patients with NSCLC between LUSC and LUAD warrants further confirmation through additional clinical trials and extensive prospective studies.

Inflammation and coagulation are closely interconnected, with fibrinogen and D-dimer as coagulation markers and molecular links in inflammation and immunity. The meta-analysis by Perisanidis et al. (49) indicated that elevated pretreatment plasma fibrinogen levels are significantly associated with reduced survival rates in patients with solid tumors. In immunotherapy, initial fibrinogen concentrations correlate with patient prognoses (50). Further research has underscored fibrinogen’s involvement in cell migration, proliferation, angiogenesis, and hematogenous metastasis (51, 52). As fibrinogen’s final degradation product, D-dimer is associated with worse progression-free survival (PFS) and OS in patients with advanced NSCLC undergoing immunotherapy with elevated levels before undergoing immunotherapy (53). The association between raised D-dimer levels and the development of deep vein thrombosis is well-documented; some researchers propose that an abnormal increase in D-dimer could influence the expression of tissue factor (TF), frequently considered to be associated with cancer metastasis and progression (54, 55). A multicenter study indicated that the neutrophil-to-lymphocyte ratio (NLR) in patients with NSCLC might significantly impact the efficacy of neoadjuvant immunotherapy (56). Although in our study, direct or indirect inflammatory indicators such as D-dimer, fibrinogen, and WBC count did not show significance in the univariate analysis of DFS, their ratios presented substantial prognostic value. The DWBCR and DFR represent the interaction between the body’s inflammatory and coagulation systems. For instance, active fibrinolysis could lead to elevated D-dimer levels and increased fibrinogen consumption, particularly during immunotherapy. This treatment phase could provoke immune-related adverse reactions such as hemolysis, the release of tumor cells into the bloodstream, and vasculitis associated with inflammatory damage. Moreover, abnormal coagulation responses could exacerbate inflammatory reactions and immune system dysfunctions, ultimately leading to reduced effects of immunotherapy (57). The capability of these ratios to improve the prognosis of neoadjuvant chemoimmunotherapy outcomes warrants further investigation. However, understanding the intricate coagulation and inflammatory mechanisms demands a multifaceted approach, integrating relevant data to evaluate the patient’s treatment responses and prognoses comprehensively.

Pulmonary function tests play a critical role in the preoperative assessment of lung cancer, essential for determining surgical viability through evaluations of respiratory function and gas exchange capacity (58). However, the predictive value of pulmonary function tests following neoadjuvant therapy remains underexplored. Despite including extensive pretreatment pulmonary function test data, only the DMVR emerged as an independent prognostic indicator in our study. The MV, a measure of lung function, efficiency, and burden, is hypothesized to be related to adverse reactions to immunotherapy. The inherent association between DMVR and its prognostic effectiveness requires further research. Typically, advanced tumor stages correlate with poorer outcomes, and the degree of pulmonary function decline before treatment can influence both treatment outcomes and prognoses. The pulmonary function data in this study were collected from patients before initiating therapy. While our research indicated that pretreatment pulmonary function does not significantly influence patient prognosis, a distinct negative correlation was observed between several indicators in the pretreatment pulmonary function tests, including VA, VC, FVC, and DLCO, and the model scores. The pulmonary immune response’s escalation following neoadjuvant therapy likely exacerbates pulmonary function. Therefore, integrating the pre- and post-treatment pulmonary function changes into the predictive model could yield more accurate forecasts for patient tumor recurrence. This hypothesis is intended to be explored in our subsequent studies.

This study has several limitations. Firstly, it was a single-center retrospective analysis involving a relatively small cohort of 175 patients, exclusively from the Chinese population, potentially introducing bias. However, carefully applying the inclusion and exclusion criteria lends a degree of representativeness to the study population. The prognostic model may be particularly predictive for Chinese patients with stage I-III NSCLC receiving neoadjuvant chemoimmunotherapy. However, the small sample size means the models’ discrimination and calibration were demonstrated through internal validation, lacking external validation. In subsequent studies, the sample size is intended to be expanded, and diverse populations are incorporated to enhance the model’s generalizability. Furthermore, although seven variables and RARS emerged as promising prognostic indicators, the categorical variables’ threshold values require validation in large-scale prospective clinical trials. Moreover, the study’s laboratory and clinical data were pretreatment measures, which might change dynamically during subsequent diagnosis and treatment, potentially affecting the accuracy of prognostic predictions. Therefore, when utilizing this model for prognostic evaluations, it is crucial to manage the timing of tests and data collection meticulously. Acknowledging these limitations illustrates the necessity for more extensive and higher-quality prospective studies.

## Conclusions

This study has identified seven prognostic factors associated with neoadjuvant chemoimmunotherapy in NSCLC, incorporating pulmonary function indicators, clinical baseline data, and postoperative pathological characteristics. These variables include smoking, T stage, MPR, CPR, DWBCR, DFR, and DMVAR. Risk models were constructed using these indicators to predict the efficacy of combined treatments and patient prognoses. Specifically, the ridge regression model from our study can assist clinicians in more accurately determining which patients have a potential risk of recurrence. Male patients with LUSC, those with concurrent COPD, and patients with a history of smoking are the likely beneficiaries of this combined therapeutic strategy. In summary, this model offers significant clinical utility.

## Data availability statement

The original contributions presented in the study are included in the article/**Supplementary Material**. Further inquiries can be directed to the corresponding authors.

## Ethics statement

The studies involving humans were approved by the Institutional Review Board of the Second Affiliated Hospital of Zhejiang University School of Medicine. The studies were conducted in accordance with the local legislation and institutional requirements. The participants provided their written informed consent to participate in this study.

## Author contributions

MZ: Writing – original draft, Visualization, Validation, Software, Formal analysis, Data curation, Conceptualization. LZ: Writing – original draft, Visualization, Validation, Software, Formal analysis, Data curation, Conceptualization. SL: Writing – review & editing, Software, Formal analysis, Data curation. ZM: Writing – review & editing, Validation, Formal analysis, Data curation. XL: Writing – review & editing, Visualization, Software, Data curation. LY: Writing – review & editing, Visualization, Software, Data curation. YY: Writing – review & editing, Validation, Data curation. KW: Writing – review & editing, Supervision, Conceptualization. PW: Writing – review & editing, Supervision, Conceptualization. WC: Writing – review & editing, Supervision, Conceptualization.

## Supplementary material

Supplementary Figure 1 Calibration evaluation of the Cox and LASSO regression models. **(A, B)**. Risk factor correlation diagrams for the **(A)** Cox regression model, and **(B)** LASSO regression model, featuring histograms of the distribution of patients’ risk scores, scatter plots of patient survival distributions, and heatmaps showing changes in variables with risk scores. The horizontal axis represents patient numbers, ranked from lowest to highest risk score; the vertical axis shows patients’ risk scores, DFS, and variables included in the model. **(C, D)**. Calibration curves for the **(C)** Cox regression model, and **(D)** LASSO regression model at the optimal calibration time point of 140 days, displaying predicted vs. actual survival status. Every 50 patients were grouped and resampled 1000 times. **(E, F)**. Nomograms for the **(E)** Cox regression model, and **(F)** LASSO regression model, based on patients’ risk scores, showing total scores and the probability of 1-year, 2-year, and 3-year DFS.

Supplementary Figure 2 Discrimination Evaluation of the Cox and LASSO regression models. **(A, B)**. The survival curves plotted based on the optimal cutoff values of 1.445, and -0.197 for the **(A)** Cox regression model, **(B)** LASSO regression model, respectively. **(C, D)**. The ROC curves for the **(C)** Cox regression model, and **(D)** LASSO regression model at 1-, 2-, 3-year disease-free survival time points, respectively. **(E, F)** PRC curves for the normalized risk scores of the **(E)** Cox regression model, and **(F)** LASSO regression, respectively. G-H. Confusion matrices for the **(G)** Cox, and **(H)** LASSO regression models, created based on the patients’ risk groups and their actual recurrence or death outcomes.
