# Supplementary material for: Pulmonary function test-related prognostic models in non-small cell lung cancer patients receiving neoadjuvant chemoimmunotherapy
Source: Front Oncol. 2024 Jun 25;14:1411436. doi: 10.3389/fonc.2024.1411436 (PMC11231186; doi:10.3389/fonc.2024.1411436)
Supplement: Supplementary file 1 [file DataSheet_1.zip › Supplementary_materials(1411436)/Supplementary tables.pdf]

Table S1. Baseline clinical characteristics of patients in our cohort (N=175)

Characteristics	No. of patients (Proportion)	P value
**Sex**		0.2448
Male	155(88.57%)	
Female	20(11.43%)	
**T stage**		**0.0171***
T1	20(11.43%)	
T2	77(44.00%)	
T3	53(30.28%)	
T4	25(14.29%)	
**N stage**		0.4054
N0	32(18.29%)	
N1	51(29.14%)	
N2	91(52.00%)	
N3	1(0.57%)	
**AJCC Stage**		0.4975
I	6(3.43%)	
II	53(30.28%)	
III	116(66.29%)	
**Pathological types**		0.1882
LUSC	123(70.29%)	
Others	52(29.71%)	
**Smoking**		**0.0065****
No	61(34.86%)	
Yes	114(65.14%)	
**CPR**		**0.0056****
No	108(61.71%)	
Yes	67(38.29%)	
**MPR**		**0.0041****
No	80(45.71%)	
Yes	95(54.29%)	
**Preoperative treatment course**		0.1448
1	2(1.14%)	
2	130(74.29%)	
3	36(20.57%)	
4	7(4.00%)	
**Surgical methods**		0.9807
Thoracoscopic surgery	156(89.14%)	
Thoracotomy	5(2.86%)	
Thoracoscopic to thoracotomy	14(8.00%)	
**Pulmonary lobectomy**		0.5932
Upper left	121(69.14%)	
Lower left	31(17.71%)	
Upper right	16(9.14%)	

Middle right	5(2.86%)	
Lower right	2(1.14%)	
**Postoperative treatment**		0.2592
No	17(9.71%)	
Yes	158(90.29%)	
**COPD**		0.9165
No	111(63.43%)	
Yes	64(36.57%)	
**Hypertension**		0.7480
No	135(77.14%)	
Yes	40(22.86%)	
**Diabetes**		0.9735
No	165(94.29%)	
Yes	10(5.71%)	
**Coronary.disease**		0.8745
No	169(96.57%)	
Yes	6(3.43%)	
**Hyperlipidemia**		0.2237
No	171(97.71%)	
Yes	4(2.29%)	
**Efficacy evaluation**		0.3454
Complete response	4(2.29%)	
Partial response	110(62.85%)	
Stable disease	60(32.28%)	
Unknown	1(0.57%)	
**Immune related pneumonia**		0.6241
No	148(81.32%)	
Yes	27(14.84%)	
**Age**		0.6264
Mean±SD	63.74±7.95	
Median[min-max]	65.00[40.00,81.00]	
**Weight**		0.3104
Mean±SD	63.73±10.24	
Median[min-max]	64.00[43.00,98.00]	
**Height**		0.9801
Mean±SD	166.17±6.51	
Median[min-max]	167.00[149.00,179.00]	
**BMI**		0.3137
Mean±SD	23.03±3.12	
Median[min-max]	23.18[15.94,33.13]	
**D-dimer**		0.0887
Mean±SD	524.46±433.41	
Median[min-max]	380.00[140.00,3620.00]	

**WBC**		0.1305
Mean±SD	7.10±2.14	
Median[min-max]	6.80[2.40,13.30]	
**Fibrinogen**		0.9086
Mean±SD	4.28±1.39	
Median[min-max]	4.10[1.99,8.77]	
**VT actual**		0.9187
Mean±SD	1.07±0.43	
Median[min-max]	0.98[0.27,2.78]	
**MV actual**		0.3738
Mean±SD	28.55±14.16	
Median[min-max]	24.97[8.09,70.82]	
**VCM actual**		0.7573
Mean±SD	3.34±0.70	
Median[min-max]	3.31[1.61,5.18]	
**FVC actual**		0.7687
Mean±SD	3.32±0.70	
Median[min-max]	3.30[1.61,5.18]	
**FEV1 actual**		0.8323
Mean±SD	2.41±0.59	
Median[min-max]	2.41[1.10,4.04]	
**FEV1/FVC actual**		0.8172
Mean±SD	72.49±8.97	
Median[min-max]	74.00[48.59,99.56]	
**PEF actual**		0.7285
Mean±SD	6.36±1.82	
Median[min-max]	6.42[2.16,13.24]	
**PEF AER**		0.5613
Mean±SD	84.84±21.65	
Median[min-max]	86.00[38.20,147.10]	
**PIF actual**		0.8526
Mean±SD	3.84±1.49	
Median[min-max]	3.62[1.42,8.37]	
**FET actual**		0.5727
Mean±SD	7.28±2.65	
Median[min-max]	6.89[1.85,15.57]	
**RV/TLC-SB actual**		0.6339
Mean±SD	42.20±8.26	
Median[min-max]	42.04[14.59,74.10]	
**RV/TLC-SB AER**		0.8627
Mean±SD	109.84±19.56	
Median[min-max]	110.20[41.70,177.30]	
**DLCOc SB actual**		0.1628
Mean±SD	6.33±1.57	

Median[min-max]	6.22[2.80,11.01]	
**DLCOc SB AER**		0.0950
Mean±SD	76.60±16.81	
Median[min-max]	76.20[32.20,127.30]	
**DLCOc/VA actual**		0.3607
Mean±SD	1.24±0.25	
Median[min-max]	1.25[0.63,2.09]	
**DLCOc/VA AER**		0.2366
Mean±SD	90.79±17.79	
Median[min-max]	90.40[48.40,176.60]	
**VA actual**		0.9401
Mean±SD	5.07±0.87	
Median[min-max]	5.04[3.36,7.86]	
**VA AER**		0.9991
Mean±SD	86.14±12.96	
Median[min-max]	85.50[60.00,137.50]	
**DAR**		0.0792
Mean±SD	8.25±6.65	
Median[min-max]	6.06[2.37,54.03]	
**DWR**		0.1437
Mean±SD	8.68±8.34	
Median[min-max]	6.48[1.94,75.42]	
**DHR**		0.0762
Mean±SD	3.16±2.62	
Median[min-max]	2.31[0.92,21.29]	
**DBR**		0.1884
Mean±SD	23.83±22.87	
Median[min-max]	17.65[5.74,217.95]	
**DWBCR**		**0.0472***
Mean±SD	77.86±63.42	
Median[min-max]	59.76[12.61,446.00]	
**DFR**		**0.0473***
Mean±SD	122.49±83.58	
Median[min-max]	98.65[35.15,592.78]	
**DVTAR**		0.0947
Mean±SD	594.59±658.44	
Median[min-max]	423.08[85.94,5048.78]	
**DMVAR**		**0.0172***
Mean±SD	24.75±26.85	
Median[min-max]	15.28[3.19,178.68]	
**DVCMAR**		0.0655
Mean±SD	166.80±151.87	
Median[min-max]	117.83[47.41,1222.97]	
**DFVCAR**		0.0633

Mean±SD	167.69±152.35	
Median[min-max]	118.81[47.41,1222.97]	
**DFEV1AR**		0.1021
Mean±SD	236.49±224.47	
Median[min-max]	162.16[58.82,1989.01]	
**DFEV1/FVCAR**		0.1488
Mean±SD	7.42±6.57	
Median[min-max]	5.22[2.15,58.83]	
**DPEFAR**		0.1286
Mean±SD	93.39±95.38	
Median[min-max]	63.68[21.48,809.84]	
**DPEF.AERR**		0.1830
Mean±SD	6.86±6.92	
Median[min-max]	4.73[1.74,63.62]	
**DPIFAR**		0.2592
Mean±SD	165.02±187.06	
Median[min-max]	114.19[30.86,1652.97]	
**DFETAR**		0.1012
Mean±SD	83.70±79.10	
Median[min-max]	56.63[18.43,477.53]	
**DRV.TLC.SBAR**		0.1321
Mean±SD	13.01±11.65	
Median[min-max]	9.57[2.10,91.44]	
**DRV.TLC.SB.AERR**		0.1411
Mean±SD	5.02±4.65	
Median[min-max]	3.61[0.79,35.95]	
**DDLCOc.SBAR**		0.1947
Mean±SD	94.34±116.41	
Median[min-max]	64.52[17.56,1292.86]	
**DDLCOc.SB.AERR**		0.2614
Mean±SD	7.65±9.70	
Median[min-max]	5.31[1.52,112.42]	
**DDLCOc.VAAR**		0.2292
Mean±SD	461.35±513.53	
Median[min-max]	330.36[95.89,5656.25]	
**DDLCOc.VA.AERR**		0.2222
Mean±SD	6.21±6.80	
Median[min-max]	4.55[1.42,74.79]	
**DVAAR**		0.0742
Mean±SD	107.62±97.86	
Median[min-max]	75.57[29.51,826.48]	
**DVA.AERR**		0.1200
Mean±SD	6.31±5.81	
Median[min-max]	4.41[1.69,53.16]	

Bold values indicate P < 0.05 and * P < 0.05, **P < 0.01. AJCC,American Joint Committee on Cancer; CPR, complete pathologic response; MPR,major pathologic response; COPD, chronic obstructive pulmonary disease BMI, body mass index; SD, standard deviation; WBC, white blood cell; VT, tidal volume; MV, minute ventilation volume; VCM, vitalcapacity max; FVC, forced vital capacity; FEV1, forced expiratory volume in one second; PEF, peak expiratory flow; AER, actual to estimate ratio; PIF,peak impiratory flow; FET, Forced Expiratory Time; RV/TLC, Residual Volume to Total Lung Capacity ratio; SB, single breath; DLCOc, Diffusing capacity of the lung for carbon monoxide corrected for haemoglobin concentration; VA, alveolar volume; DXXR, D-dimer/XX ratio, and XX indicate the continuous variables listed in the table.

Table S2. Comparative analysis of discrimination indexes of the three models in our study

Indexes	Cox regression model	P-value	LASSO regression model	P-value	Ridge regression model
**ROC AUC (95% CI)**					
1-year DFS	0.756 (0.663, 0.850)		0.725 (0.630, 0.819)		0.743 (0.649, 0.837)
2-years DFS	0.807 (0.664, 0.951)		0.811 (0.6774, 0.945)		0.814 (0.684, 0.943)
3-years DFS	0.825 (0.646, 1.003)		0.723 (0.506, 0.940)		0.786 (0.598, 0.973)
**DCA AUC**					
1-year DFS	0.007		0.010		0.012
2-years DFS	0.050		0.074		0.071
3-years DFS	0.089		0.095		0.131
**IDI[$] (95% CI)**					
1-year DFS	0.012 (-0.039, 0.047)	0.479	0.009 (-0.013, 0.033)	0.379	
2-years DFS	0.044 (-0.045, 0.146)	0.259	0.003 (-0.059, 0.062)	0.922	
3-years DFS	0.034 (-0.162, 0.224)	0.510	0.032 (-0.074, 0.118)	0.332	
**Continous-NRI[$] (95% CI)**					
1-year DFS	0.253 (0.030, 0.482)	**0.025**	0.185 (-0.169, 0.403)	0.293	
2-years DFS	0.269 (-0.242, 0.628)	0.154	0.031 (-0.0565, 0.472)	0.926	
3-years DFS	0.157 (-0.812, 1.009)	0.613	0.374 (-0.877, 1.196)	0.422	
**Median improvement[$] (95% CI)**					
1-year DFS	0.028 (0.005, 0.078)	**0.014**	0.009 (-0.008, 0.041)	0.267	
2-years DFS	0.092 (-0.041, 0.192)	0.164	0.000 (-0.050, 0.058)	0.886	
3-years DFS	0.029 (-0.133, 0.236)	0.732	0.030 (-0.050, 0.100)	0.488	

$ compared with the ridge regression model. Bold values indicate P < 0.05. LASSO, least absolute shrinkage and selection operator; ROC, receiver operating characteristic; AUC, area under the curve; DCA, decision curve analysis; NRI, net reclassification index; IDI, integrated discrimination improvement.
