# Supplementary figures and images for: Pulmonary function test-related prognostic models in non-small cell lung cancer patients receiving neoadjuvant chemoimmunotherapy
Source: Front Oncol. 2024 Jun 25;14:1411436. doi: 10.3389/fonc.2024.1411436 (PMC11231186; doi:10.3389/fonc.2024.1411436)

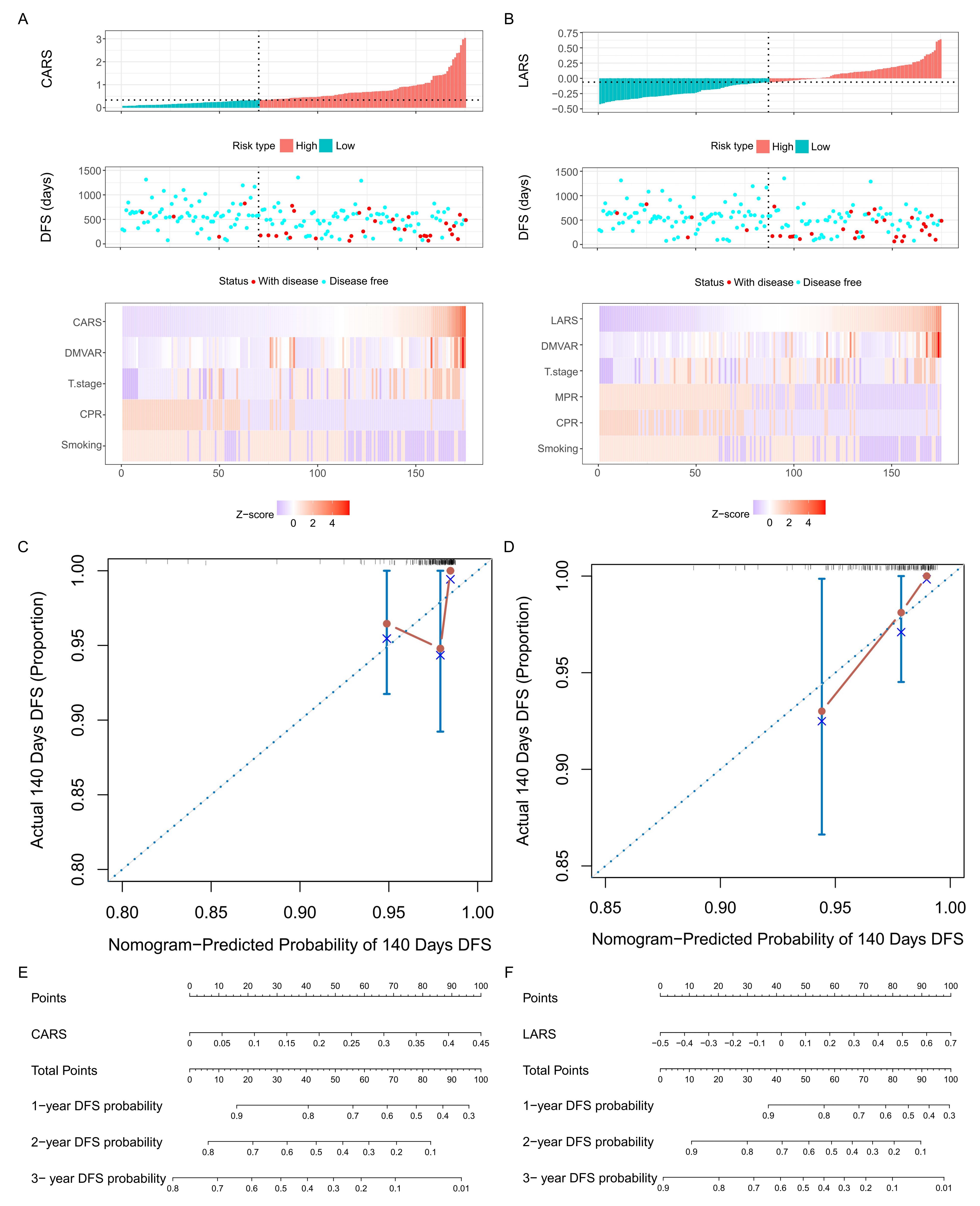

Supplement: Supplementary file 1 [file DataSheet_1.zip › Supplementary_materials(1411436)/figureS1.jpg]

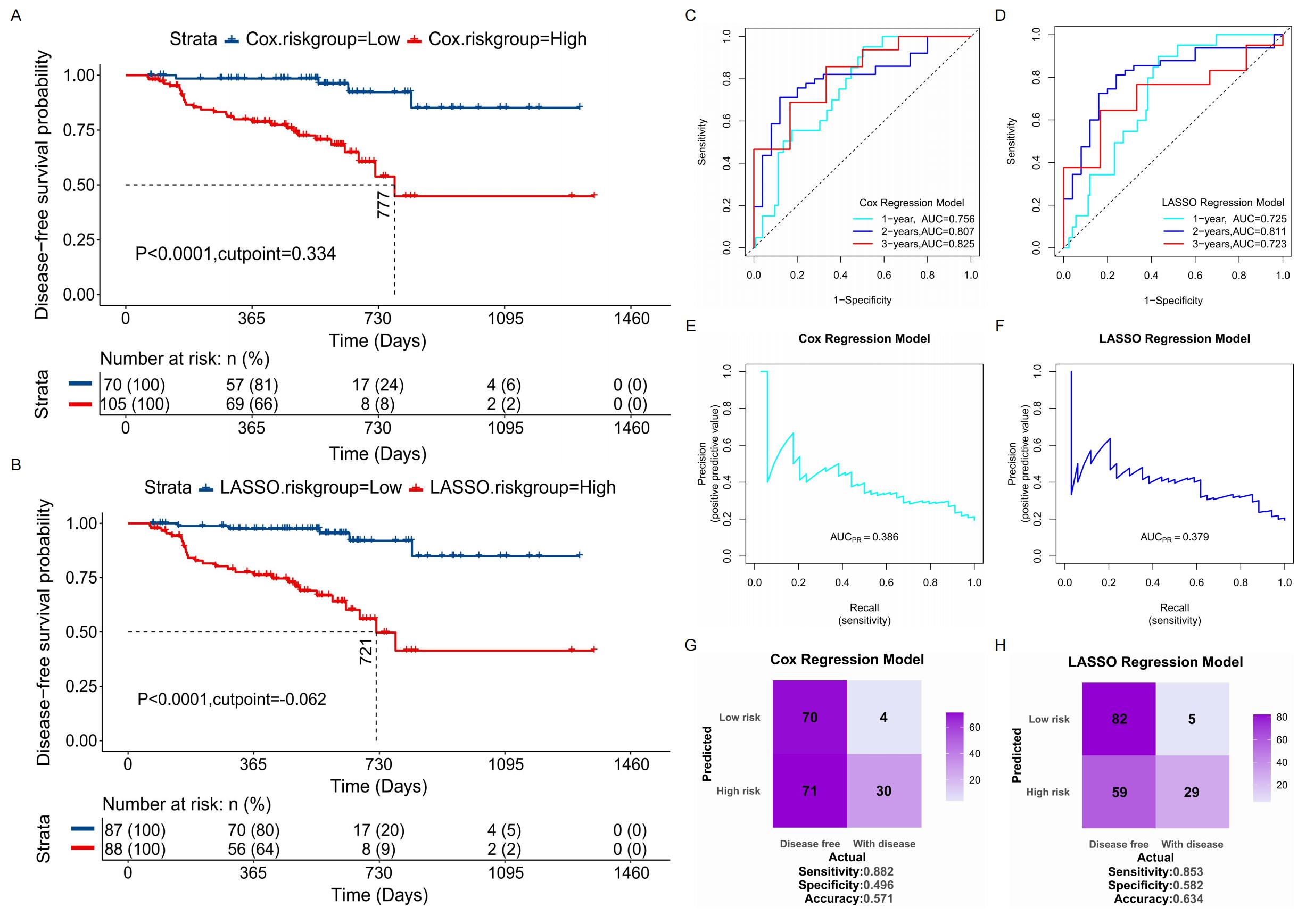

Supplement: Supplementary file 1 [file DataSheet_1.zip › Supplementary_materials(1411436)/figureS2.jpg]
